# Therapeutics of a Sea Voyage

**Published:** 1903-03

**Authors:** R. Roxburgh

**Affiliations:** Honorary Physician, Weston-super-Mare Hospital and Dispensary.


"*Scire est nescire, nisi id me*
*Scire alius sciret.*"

MARCH, 1903.

### THERAPEUTICS OF A SEA VOYAGE.

BY

R. Roxburgh, M.B. F.R.C.S. Ed.,

*Honorary Physician, Weston-super-Mare Hospital and Dispensary.*

Up till about the sixth or seventh decade of the nineteenth century the "sea voyage" held a definite place in the list of favourite therapeutic measures. Amongst others, the distinguished Dr. Andrew Coombe—himself a sufferer from chronic phthisis — warmly advocated its adoption in the treatment of that disease, and in doing so spoke from his own personal experience. In recent years its popularity has markedly declined, and it has become a comparatively rare prescription.

The principal cause of this is to be found in the altered conditions of ocean travel. Passengers are no longer conveyed to India, America, or Australasia in sailing-ships, which take months of varied experience on the wide seas to reach their destination, but are hurried thither in half or a quarter of the time in crowded steamers, whose dates of arrival can be calculated with mathematical precision. This change has been called for by the advance of trade and civilisation. Time is the most precious of all commodities, and, other things being equal, the fastest vessels will always be the most favoured. But from the point of view of the health-seeker there can be no comparison between the two kinds of transit.

The liability to sea-sickness, the terror of so many persons, is infinitely less in a sailing-ship than in a steamer. The constant vibration of the steamer certainly predisposes to sickness, and it is impossible, even under the best conditions, to wholly eliminate the disagreeable emanations from the engine-room, the disturbing noise and throb of machinery, or the falling of soot on the deck. Above all, the steamer pursues her way *relentlessly*, irrespective of contrary winds and currents, so that the inco-ordinate movement of combined pitching and rolling, with the thump of the propeller, are trying even to the most seasoned traveller. How different is the passive and yielding movement of the sailing-ship. By the very laws which govern her propulsion she cannot ruthlessly attack a head sea. Her navigation depends on the constancy of the Trade winds and the ocean currents, and, by taking full advantage of these, she usually enjoys a wind more or less abaft the beam, and her movements are comparatively gentle and regular, while in calm weather, with all canvas set, she slips along with a delicious smoothness comparable only to skating or flying. In a long voyage by steamer, a "bad sailor" may suffer so continuously as to be brought into a state of dangerous asthenia, but in a sailing-vessel sickness is but transient.

In the course of a recent tour round the world I had occasion to make such observations as these. I had engaged a berth in a steamship bound for Australia, because I could not hear of a sailing-ship intended for passenger service. For, although the harbours of the world are crowded with splendid sailing-craft, hardly any of these are fitted up for passengers, or carry a doctor.

Finding, however, that Messrs. Devitt and Moore's fine three-masted clipper *Illawarra*, of 3,700 tons burthen, Captain Maitland, was furnished with good accommodation, and was to leave England for Melbourne on September 25th, 1901, I cancelled my engagement, and took a berth in the *Illawarra*. This well-appointed iron ship carries about thirty young cadets who are trained for the merchant service, and are systematically taught the theory and art of navigation by a special nautical Tutor, as well as by the Captain and Officers; and they also assist the crew in working the ship. There is berth accommodation for twenty-two passengers; but, as we carried less than a dozen, everyone had a self-contained cabin. The saloon and passengers' and Captain's cabins occupied the poop, while the cadets' and officers' quarters were amidships. Dr. Hall, of the Birmingham School, was Medical Officer.

We left Plymouth on the 25th September, and encountered rough weather in the Channel. For several days most of the passengers felt uncomfortable; but by the end of the week we had passed the Bay of Biscay and were off Finisterre, and all had fully recovered. In another week we were bowling along with the North-east Trades in exquisite weather, with all ports and hatches open. On the 24th October we "crossed the line." Even when sailing through tropical latitudes the heat was almost constantly modified by a gentle, cool breeze, blowing by night and day, while frequent short-lived showers of tropical density still further freshened the atmosphere. It was not till we had reached 23° south of the Equator that we experienced the "doldrums," and these were soon over. The first fortnight of November was marked by a daily fall of temperature, which required a rapid resumption of warm clothing, and by the 15th of that month we were in the "roaring forties"—the strong, cold, southerly winds that prevail between the 40th and 50th parallel south of the Equator. Latitude 40° in the South Atlantic, corresponds in temperature to about 51° in the North Atlantic, because the Northern Hemisphere, consisting mostly of land, retains the solar heat, while the Southern Hemisphere is chiefly ocean. On November 20th we were 500 miles south of the Cape of Good Hope. On December 4th in latitude 42° 45′ S. (corresponding to that of the Riviera, north of the Equator), the temperature was 32° F., and we were running before a tearing south-west wind, with frequent hail and snow squalls, and shipping large seas—a contrast indeed to the weather of a few weeks before; and this was summer! Similar weather lasted till Australia was reached on the 22nd December. On the 23rd the *Illawarra* lay at her wharf in Melbourne, having sailed 14,000 miles in 88 days at an average speed of 159 knots a day.

In Melbourne another acute change of temperature awaited us. On Christmas Day, which occurs at Midsummer, the heat and the dust were most oppressive, and on Boxing Day the temperature at noon was 110° F. in the shade! The only relief was to spend the day in bathing, facilities for which are provided at seaside resorts easily accessible from town.

Therapeutically, what is to be said for and against such a voyage as this? In incipient phthisis much benefit may be looked for from the constant inhalation during waking hours of the purest possible atmosphere, the increased appetite engendered by the sea voyage and the fresh breezes, the large amount of sunlight in tropical and sub-tropical regions, and the easy forms of exercise cultivated on board ship. In advanced cases the violent changes of temperature, and the absence of home comforts are an absolute bar to this treatment. The gain to the pulmonary subject, too, must be discounted by the difficulty of securing perfect ventilation below. In rough weather all ports have to be closed, and a sailing-vessel has not the electric fans which are so inestimable a boon in the luxurious modern liner. Improved ventilation is the one great desideratum in designing a passenger-bearing craft, and there is no reason why ingenuity should not overcome the difficulty. It may be remarked that the homeward voyage from Australia is altogether more suitable for the invalid than the outward. The ship then takes a more northerly direction from Melbourne, soon emerges from the "roaring forties," steers for Natal, passes round the

Cape with the Agulhas and Southern Atlantic currents, and calls at St. Helena. The return voyage is longer but more agreeable; and as the *Illawarra* generally carries home a cargo mainly of wool, she is more buoyant than with the heavy mixed cargo with which she sails to the Colonies.

In convalescence from acute illness, in nerve exhaustion from over-work or any other cause, and in the anæmia and enfeeblement of rapid adolescence ("outgrowing his strength"), no treatment could possibly be better or as good as the long sea voyage described. Such absolute repose of body and mind can never be obtained at any health resort at home or abroad. The absence of a daily newspaper and the excitements of the telegraph and post, the freedom from all petty anxieties and social worries, the perfect physical rest, the soporific effects of the air, and the cradle-like movement of the great ship, the unrivalled opportunity for general reading, the firsthand contact with Nature in some of her grandest aspects, the ever-changing moods of the ocean, the marvellous flight of the albatross, the ceaseless escort of different kinds of birds, the shoals of flying fish, the spouting of whales, the speaking of other ships, and the interest centering in the ship herself as a kind of personality, and her behaviour in various circumstances: all these are wholesome and recuperant features in an environment which reduces life to its primal elements, and banishes fatigue of the nerve centres and of the organic processes.

In the neurasthenia accompanying prolonged dyspepsia the voyage is contra-indicated. The delicate dietary and the supplies of fresh milk required in such conditions are not available; and although the food on board the *Illawarra* was most excellent of its kind, and attractively cooked and served (a number of live sheep, pigs and poultry were carried, and fresh meat was supplied daily), yet a certain amount of monotony was unavoidable—hardly appreciable by a healthy stomach, but unfavourable for the dyspeptic.

What about the general monotony of the voyage? someone asks. To persons whose happiness depends mostly on society, the circle of officers and passengers is felt, perhaps, to be too restricted; but to the lover of books and Nature there is no monotony except during "easting down" in the "roaring forties," when reading is prohibited by cold winds and soaking decks, and the sea is too sullen and angry to be lovable. Moreover, there is no lack of amusement—concerts, games, etc. As before mentioned, however, the return voyage is under happier auspices as regards weather.

Three months is a long period to be out of sight of land, and there is no doubt that, from the passenger's point of view, the charms of the voyage would be enormously enhanced by calling at such interesting spots as Madeira, Tristan d'Acunha, St. Paul. But this would be "ocean-yachting," and is inconsistent with the direct and quick passage exacted by the requirements of trade.

In a future paper I hope to touch on a few more matters of interest from a medical standpoint observed during succeeding travels.

